# Frailty, but not cognitive impairment, improves mortality risk prediction among those with chronic kidney disease—a nationally representative study

**DOI:** 10.1186/s12882-024-03613-y

**Published:** 2024-05-22

**Authors:** Jingyao Hong, Nadia M. Chu, Samuel G. Cockey, Jane Long, Nicolai Cronin, Nidhi Ghildayal, Rasheeda K. Hall, Megan Huisingh-Scheetz, Jennifer Scherer, Dorry L. Segev, Mara A. McAdams-DeMarco

**Affiliations:** 1 Department of Surgery, NYU Grossman School of Medicine and NYU Langone Health, New York, NY USA; 2 Department of Surgery, Johns Hopkins University, Baltimore, MD USA; 3 Department of Medicine, Duke University School of Medicine, Durham, NC USA; 4 Durham Veterans Affairs Medical Center, Durham, NC USA; 5 Department of Medicine, University of Chicago, Section of Geriatrics and Palliative Medicine, Chicago, IL USA; 6 Department of Population Health, NYU Grossman School of Medicine, New York, NY USA

**Keywords:** Frailty, CKD, Cognitive impairment, Mortality

## Abstract

**Background:**

Though older adults with chronic kidney disease (CKD) have a greater mortality risk than those without CKD, traditional risk factors poorly predict mortality in this population. Therefore, we tested our hypothesis that two common geriatric risk factors, frailty and cognitive impairment, and their co-occurrence, might improve mortality risk prediction in CKD.

**Methods:**

Among participants aged ≥ 60 years from National Health and Nutrition Examination Survey (2011–2014), we quantified associations between frailty (physical frailty phenotype) and global/domain-specific cognitive function (immediate-recall [CERAD-WL], delayed-recall [CERAD-DL], verbal fluency [AF], executive function/processing speed [DSST], and global [standardized-average of 4 domain-specific tests]) using linear regression, and tested whether associations differed by CKD using a Wald test. We then tested whether frailty, global cognitive impairment (1.5SD below the mean), or their combination improved prediction of mortality (Cox models, c-statistics) compared to base models (likelihood-ratios) among those with and without CKD.

**Results:**

Among 3,211 participants, 1.4% were cognitively impaired, and 10.0% were frail; frailty and cognitive impairment co-occurrence was greater among those with CKD versus those without (1.2%vs.0.1%). Frailty was associated with worse global cognitive function (Cohen’s d = -0.26SD,95%CI -0.36,-0.17), and worse cognitive function across all domains; these associations did not differ by CKD (p_interactions_ > 0.05). Mortality risk prediction improved only among those with CKD when accounting for frailty (p_[likelihood ratio test]_ < 0.001) but not cognitive impairment.

**Conclusions:**

Frailty is associated with worse cognitive function regardless of CKD status. While CKD and frailty improved mortality prediction, cognitive impairment did not. Risk prediction tools should incorporate frailty to improve mortality prediction among those with CKD.

**Supplementary Information:**

The online version contains supplementary material available at 10.1186/s12882-024-03613-y.

## Background

Chronic kidney disease (CKD) affects an estimated 13% of the population [1], and has a disproportionately higher prevalence among older adults [2]. It is currently the 10th leading cause of death in the United States, with an age-adjusted death rate of about 12.7 per 100,000 U.S. standard population [3]. A diagnosis of CKD is known to confer a 36% increase in mortality risk [4, 5], independent of other cardiovascular risk factors [6]. Even mild decreases in kidney function are associated with significantly increased mortality risk [7, 8].

Frailty and cognitive impairment are two geriatric risk factors common in older adults with CKD [9–11]. Physical frailty is a syndrome [12–14] distinct, but related to comorbidity and disability [15], occurring in approximately 10% to 15% of community-living older adults [14, 16]. Mild cognitive impairment affects approximately 16% to 20% of older adults [17], and is increasingly recognized as a prodromal phase of many types of dementias  [17–21]. Studies have demonstrated a frailty-cognition link, collectively suggesting that frailty is associated with lower levels and steeper declines in cognitive function, and vice-versa [22–27]. However, it remains unclear whether CKD exacerbates the relationship between frailty and cognitive function.

Risk prediction is of critical importance in an era of personalized medicine, where patients of the highest risk can be directed toward tailored therapeutic intervention, high touch care, and prevention measures, or selected for clinical trials, health care policy research, or counseling in a more evidence-based manner [9]. Prediction models with traditional risk factors poorly predict mortality for patients with CKD. Models that only use factors such as demographics, lifestyle, and kidney function to predict mortality in patients with CKD have C-statistics ranging from 0.71 to 0.81 [28–30]. Therefore, efforts to assess the predictive ability of novel risk factors are needed to improve the accuracy of prediction survival models in CKD [9].

Frailty and cognitive impairment are both predictive of adverse outcomes among the general older adult population [15, 31–35], and among those with end-stage kidney disease (ESKD)  [22, 36–45]. However, it remains unclear whether these two common, interrelated risk factors together improve the predictive ability of mortality risk among those with CKD, as they have been shown to do in other populations [46]. This question is of critical importance given that both frailty and cognitive impairment are dynamic conditions which are potentially modifiable or preventable [47–51]; thus, providing another optimistic avenue for therapeutic intervention or prevention measures for those most vulnerable.To test whether the association between frailty and cognitive function differs by CKD and to evaluate their predictive ability of mortality among those with and without CKD, we leveraged the National Health and Nutrition Examination Survey (NHANES, 2011–2014), a nationally-representative cross-sectional study designed to assess the health and nutritional status of the U.S. civilian noninstitutionalized resident population [52]. Our goals were to: (1) quantify the association between frailty and global and domain-specific cognitive function, (2) test whether these associations differed between those with versus without CKD, and (3) test whether the separate and co-occurrence of frailty and cognitive impairment improve mortality risk prediction among those with versus without CKD.

## Methods

### Study design

We leveraged 3,211 participants aged 60 years and older from NHANES (2011–2014) with measures of serum creatinine (for eGFR calculation), at least one assessment of cognitive function, and a measure of frailty, as described below. Study design of the 2011–2014 NHANES survey cycle was described elsewhere [53, 54]. Two cross-sectional studies were conducted separately during cycle 2011–2012 and cycle 2013–2014, with one round of interview and examination conducted for each participant. We combined the two cycles to produce 2011–2014 estimates. In our study, 1,523 participants were from cycle 2011–2012, and 1,688 were from 2013–2014.Participants’ demographic information, including education (high school degree or higher), and health status were collected either through direct measurement or via self-report, including: body mass index (BMI) (weight divided by height squared); depressive symptoms (Patient Health Questionnaire [PHQ-9] ≥ 10); hypertension (systolic blood pressure [SBP] ≥ 130 mmHg, diastolic blood pressure [DBP] ≥ 80 mmHg, or reported current use of antihypertensive medication); diabetes (fasting blood glucose level ≥ 126 mg/dL, non-fasting glucose level ≥ 200 mg/dL, reported history of diabetes or current use of medications for diabetes or high blood sugar); anemia (hemoglobin < 12 g/dL in males, hemoglobin < 11 g/dL in females, or reported taking treatment for anemia in the past 3 months). History of coronary heart disease (CHD), myocardial infarction (MI), stroke, and smoking (smoked at least 100 cigarettes in life). were collected by self-report using a questionnaire.

### CKD

Serum creatinine and albuminuria (ACR) were measured by laboratory tests. Serum creatinine was used to calculate the estimated glomerular filtration rate (eGFR) with the CKD-EPI equation [55]. We defined CKD based on previously published guidelines (eGFR < 60 mL/min/1.73m^2^ or albuminuria ≥ 30 mg/g) [56].

### Cognitive function

We defined global and domain-specific cognitive functions using the objective measures available in NHANES at each cycle (2011–2014). Measures included word list learning trials from the Consortium to Establish a Registry for Alzheimer’s Disease (CERAD) battery for immediate recall (CERAD-WL) and delayed recall (CERAD-DL) [57], the Animal Fluency (AF) test for verbal fluency [58, 59], and the Digit Symbol Substitution Test (DSST) for executive function and processing speed [60–62]. The CERAD word list learning task for immediate recall (CERAD-WL) and delayed recall (CERAD-DR) examines the ability to recall newly learned information and delayed memory. Specifically, 10 words were read aloud by the participant, immediately followed by 3 consecutive recalls [58, 63, 64]. The delayed recall of all 10 words occurred after the AF and DSST assessments, approximately 8–10 min from the start of the word learning trials [58, 63, 64]. The AF is a verbal fluency test that assesses semantic memory, where participants were asked to name aloud as many animals as possible in 1 min [58, 59]. The DSST is a paper-and-pencil cognitive test that primarily assesses attention and processing speed [60], but is also linked to executive functioning [60–62]. The examination was conducted using a paper form that has a key at the top containing 9 numbers paired with symbols. Participants copy the matching symbol in boxes that adjoin the numbers in 2 min. For each cognitive test, 1 point was given for each correctly recalled, named, or matched response, with a higher score reflecting better cognitive function [58, 63, 64].

All objective tests were then standardized to a mean of 0 and standard deviation (SD) of 1 and averaged into a global cognitive composite score, as described in prior studies [65]. Global cognitive impairment was defined as having a global cognitive composite score of 1.5 SD below the mean.

### Frailty

Frailty was operationalized using an adapted, 5-item physical frailty phenotype [32] comprising weight loss, low grip strength, exhaustion, slow gait speed, and low physical activity (Table S1). Participants were scored as 0 or 1 for the absence or presence of each criterion. Scores were then summed for each participant (range: 0 to 5), where participants with values of 3 or greater were defined as frail, and those with values of 2 or less were not frail.

### Descriptive statistics

We summarized the distributions of participant characteristics by CKD status, generating means and standard deviations for normally distributed continuous variables, medians and inter-quartile ranges (IQRs) for non-normally distributed continuous variables, and proportions for binary or categorical variables. Mobile examination center sample weights were applied in all analyses to produce estimates representative of the U.S. noninstitutionalized civilian resident population [66].

### Frailty, CKD, and cognitive function

We used adjusted linear regression models to assess the standardized difference in mean cognitive function by frailty (Cohen’s d), where effect sizes of 0.2 SD were considered small, 0.5 SD medium, and 0.8 SD large. To assess whether associations differed between those with and without CKD, we tested the interaction between CKD and frailty using a Wald test. Models were adjusted for potential frailty and cognitive function confounders, including age, sex, race, education, BMI, depression, hypertension, diabetes, CHD, stroke, MI, anemia, and smoking. The mobile examination center sample weights were accounted for to generate nationally-representative estimates.

### Predictive ability of CKD, frailty, and cognitive impairment on mortality risk

We quantified the impact of CKD, frailty, and cognitive impairment, individually and in combination, on all-cause mortality risk using Cox proportional hazard models, and compared cumulative incidence functions. Proportional hazard assumptions were confirmed by visual inspection of the complementary log–log plots and Schoenfeld residuals. We then compared likelihood ratio tests, Harrell’s C-stat, Akaike’s information criterion (AIC) and Bayesian information criterion (BIC) for these models to a base model adjusting for age, sex, race, education, hypertension, diabetes, CHD, MI, stroke, anemia, and smoking. Higher C-stats, lower AIC and lower BIC values suggest better fit of the model. In order to perform these comparisons, the analyses were not weighted.

### Statistical analysis

All statistical analysis was conducted using Stata version 16, and we used a statistical significance cut-off of α < 0.05.

## Results

### Participant characteristics

Among the study population, 16.8% were 80 years or older, 78.5% were Non-Hispanic White, 54.2% were female, 81.5% had a high school diploma or higher education, and 37.5% had CKD (Table 1). Median scores for cognitive tests were 20 (IQR: 17–23) for immediate recall (CERAD-WL), 6 (IQR: 5–8) for delayed recall (CERAD-DR), 18 (IQR: 14–21) for verbal fluency (AF), and 53 (IQR:41–64) for executive function and processing speed (DSST).

**Table 1:** Characteristics of participants aged 60 years and older with and without chronic kidney disease (CKD) from the National Health and Nutrition Examination Survey (NHANES) (2011–2014) (*n* = 3,211). Proportions (%) are presented unless otherwise indicated, accounting for NHANES sampling weights. eGFR was calculated using serum creatinine and the CKD-EPI equation; CKD was defined as eGFR < 60 mL/min/1.73m^2^ or ACR ≥ 30 mg/g. Frailty was categorized using an adapted, 5-item physical frailty phenotype. Abbreviations: CHD, coronary heart disease; MI, myocardial infarction; BMI, body mass index; IQR, interquartile range, CERAD-WL, the Consortium to Establish a Registry for Alzheimer’s Disease word learning subtest immediate recall module; CERAD-DR, the Consortium to Establish a Registry for Alzheimer’s Disease word learning subtest delayed recall module; AF, the Animal Fluency test; DSST, the Digit Symbol Substitution test

Characteristic	Overall (*N* = 3,211)	No CKD (*N* = 2,050)	CKD (*N* = 1,161)
Age (years)			
60–69	53.7	63.6	33.6
70–79	29.5	26.9	34.8
≥80	16.8	9.5	31.6
Race			
Mexican American	3.8	3.9	3.5
Other Hispanic	3.8	3.9	3.5
Non-Hispanic White	78.5	78.7	77.9
Non-Hispanic Black	8.4	7.8	9.7
Non-Hispanic Asian	3.9	4.3	3.2
Other	1.7	1.5	2.2
Female	54.2	53.1	56.6
Education ≥ 12 years	81.5	84.4	75.7
Hypertension	75.5	70.2	86.2
Diabetes	23.2	18.2	33.4
CHD	10.0	7.2	15.7
MI	8.9	6.5	13.6
Stroke	7.7	5.3	12.5
Anemia	7.0	3.9	13.3
Ever smoking	50.1	49.1	52.1
BMI (kg/m^2^), median (IQR)	27.9 (7.4)	27.7 (7.1)	28.2 (7.9)

### Frailty and cognitive function among those with and without CKD

**Fig. 1:**
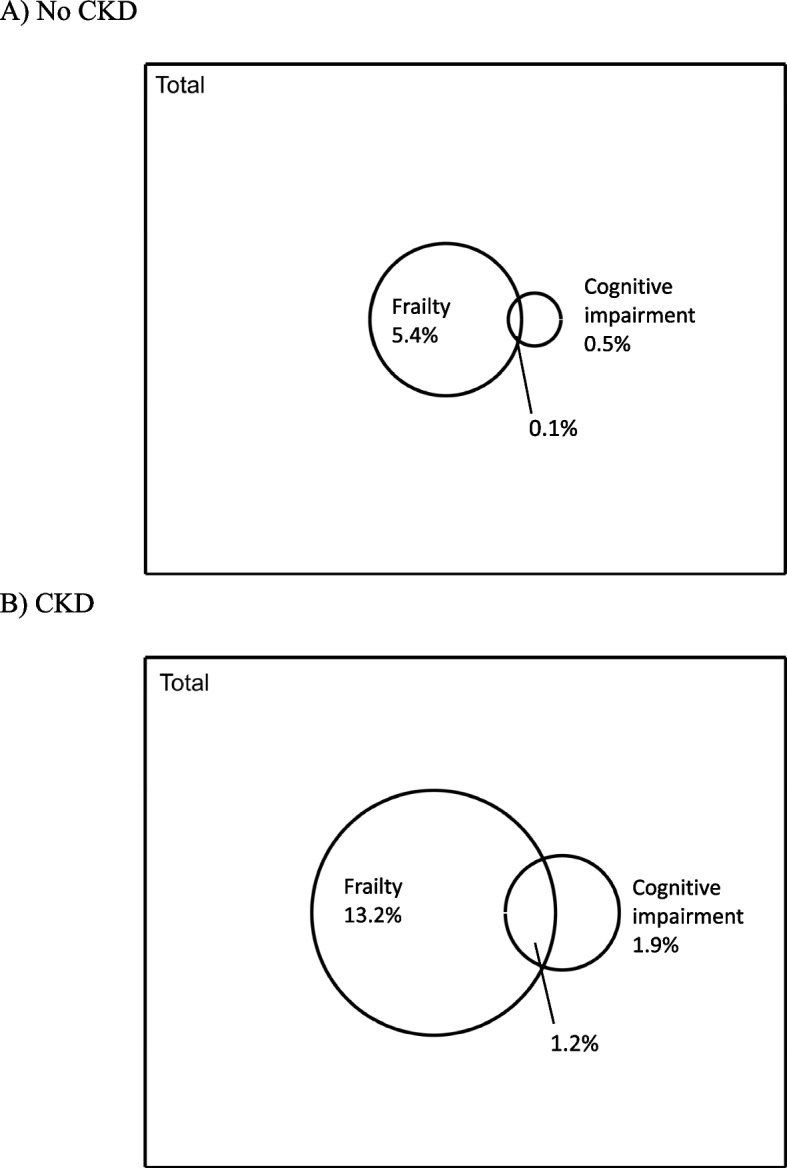
Burden of frailty and global cognitive impairment among participants aged 60 years and older in the National Health and Nutrition Examination Survey (NHANES) (2011–2014) **A**) without CKD (*n* = 2,050) and **B**) with CKD (*n* = 1,161). NHANES sampling weights were accounted for to obtain nationally-representative estimates. EGFR was calculated using serum creatinine and the CKD-EPI equation; CKD was defined as eGFR < 60 mL/min/1.73m^2^ or ACR ≥ 30 mg/g. Frailty was categorized using an adapted 5-item frailty phenotype. Cognitive impairment was defined as global cognitive function (average score of 4 objective cognitive tests) less than 1.5 SD below the mean

Those with CKD had a greater prevalence of frailty (13.2% vs. 5.4%), cognitive impairment (1.9% vs. 0.5%), and co-occurrence of frailty and cognitive impairment (1.2% vs. 0.1%) (Fig. 1). Unadjusted associations are reported in Table S2. After adjustment, frailty was associated with worse global cognitive function (Cohen’s d = -0.26 SD, 95%CI: -0.36, -0.17); however, this association did not differ for those with versus without CKD (p for interaction = 0.58) (Table 2). Additionally, frailty was associated with worse cognitive function across all domains, including immediate recall (Cohen’s d = -0.24, 95%CI: -0.37, -0.12), delayed recall (Cohen’s d = -0.18, 95%CI: -0.28, -0.08), verbal fluency (Cohen’s d = -0.32, 95%CI: -0.44, -0.19), and executive function and processing speed (Cohen’s d = -0.44, 95%CI: -0.60, -0.28); none of those associations differed for those with versus without CKD (all p for interactions > 0.5) (Table 2). When cognitive function was measured by self-perceived cognitive decline (Table S3); and when frailty was operationalized using an alternative, 4-item physical frailty phenotype (Table S5), all p for interactions remained > 0.05.

**Table 2:** Association between frailty, chronic kidney disease (CKD) and global and domain-specific cognitive function among participants aged 60 years and older from the National Health and Nutrition Examination Survey (NHANES) (2011–2014) (*n*=3,211). Cognitive test scores were standardized to a mean of 0 and standard deviation of 1. Global cognitive function was defined as average score of all 4 objective cognitive tests. Models were adjusted for age, sex, race, education, hypertension, diabetes, coronary heart disease, myocardial infarction, stroke, anemia, and smoking. NHANES sampling weights were accounted for in linear regression analyses to obtain nationally-representative estimates. EGFR was calculated using serum creatinine and the CKD-EPI equation; CKD was defined as eGFR < 60 mL/min/1.73m^2^ or ACR ≥ 30 mg/g. Frailty was categorized using an adapted 5-item physical frailty phenotype. Abbreviations: CI, confidence interval; CERAD-WL, the Consortium to Establish a Registry for Alzheimer’s Disease word learning subtest immediate recall module; CERAD-DR, the Consortium to Establish a Registry for Alzheimer’s Disease word learning subtest delayed recall module; AF, the Animal Fluency test; DSST, the Digit Symbol Substitution test

	Overall	No CKD	CKD	
	Mean (95% CI)	Mean (95% CI)	Mean (95% CI)	*p* [interaction]
Global cognitive function				
Not frail	0 (reference)	0 (reference)	0 (reference)	
Frail	-0.26 (-0.36, -0.17)	-0.21 (-0.37, -0.04)	-0.30 (-0.45, -0.15)	0.48
Immediate recall (CERAD-WL)				
Not frail	0 (reference)	0 (reference)	0 (reference)	
Frail	-0.24 (-0.37, -0.12)	-0.25 (-0.49, -0.01)	-0.23 (-0.38, -0.07)	0.90
Delayed recall (CERAD-DR)				
Not frail	0 (reference)	0 (reference)	0 (reference)	
Frail	-0.18 (-0.28, -0.08)	-0.16 (-0.38, 0.06)	-0.19 (-0.41, 0.03)	0.86
Verbal fluency (AF)				
Not frail	0 (reference)	0 (reference)	0 (reference)	
Frail	-0.32 (-0.44, -0.19)	-0.29 (-0.50, -0.08)	-0.33 (-0.48, -0.18)	0.76
Executive function & processing speed (DSST)				
Not frail	0 (reference)	0 (reference)	0 (reference)	
Frail	-0.44 (-0.60, -0.28)	-0.38 (-0.59, -0.18)	-0.48 (-0.68, -0.28)	0.47

### Frailty and cognitive impairment risk prediction

#### Mortality risk prediction among those without CKD

Among those without CKD, neither frailty (HR = 1.14, 95%CI: 0.44, 3.00; C-stat = 0.784) nor cognitive impairment (HR = 0.88, 95%CI: 0.12, 6.69; C-stat = 0.782) were associated with increased mortality risk (Table 3). Furthermore, neither frailty nor cognitive impairment improve risk prediction of mortality when comparing them individually (frailty only: p for likelihood ratio test [LR] = 0.79; cognitive impairment only: p for LR = 0.90) or in combination (p for LR = 0.95) to the base model.

**Table 3:** Predictive ability of CKD, frailty, and cognitive impairment on risk of all-cause mortality among participants aged 60 years and older from the National Health and Nutrition Examination Survey (NHANES) (2011–2014) (*n*=2,663). Predictive ability of chronic kidney disease (CKD), frailty, and/or cognitive impairment on risk of mortality was assessed by comparing likelihood ratio tests for Cox proportional hazards models that added these variables individually and in combination to a base model that adjusted for age, sex, race, education, hypertension, diabetes, coronary heart disease, myocardial infarction, stroke, anemia, and smoking. Higher C-stats, lower AIC and lower BIC values suggest better fit of the model. EGFR was calculated using serum creatinine and the CKD-EPI equation; CKD was defined as eGFR < 60 mL/min/1.73m^2^ or ACR ≥ 30 mg/g. Frailty was categorized using an adapted, 5-item frailty phenotype. Cognitive impairment (CI) was defined as global cognitive function (average score of all 4 objective cognitive tests) less than 1.5 standard deviations below the mean. Abbreviations: HR, hazard ratio, CI, cognitive impairment; LR, likelihood ratio

Model	HR (95% Confidence Interval)	C-statistic	AIC	BIC	p_[LR]_^*^
No CKD (*n*=1,755)					
Base model (ref)	1 (reference)	0.782	646.9	734.4	(ref)
Frailty model	1.14 (0.44, 3.00)	0.784	648.8	741.8	0.79
CI model	0.88 (0.12, 6.69)	0.782	648.9	741.9	0.90
Frailty + CI model	Frailty: 1.15 (0.44, 3.03)	0.784	650.8	749.3	0.95
	CI: 0.86 (0.11, 6.57)				
CKD (*n*=908)					
Base model (ref)	1 (reference)	0.722	1241.8	1318.8	(ref)
Frailty model	2.74 (1.74, 4.32)	0.751	1226.7	1308.5	**<0.001**
CI model	1.73 (0.80, 3.77)	0.730	1242.1	1323.9	0.19
Frailty + CI model	Frailty: 2.70 (1.71, 4.25)	0.754	1227.4	1314.0	**<0.001**
	CI: 1.59 (0.74, 3.45)				

^*^p[LR] *p*-value for likelihood ratio test

#### Mortality risk prediction among those with CKD

Participants were followed until death, for a median follow-up time of 30 months (Table S5). Among those with CKD, frailty (HR = 2.74, 95%CI: 1.74, 4.32; C-stat = 0.751) was associated with increased mortality risk, but cognitive impairment was not (HR = 1.73, 95%CI: 0.80, 3.77; C-stat = 0.730) (Table 3). Additionally, frailty significantly improved the prediction of mortality (p for LR < 0.001) compared to the base model, while cognitive impairment did not (p for LR = 0.19).

## Discussion

In this nationally representative study of older adults aged 60 years and older, we found that those with CKD had a greater prevalence of frailty (13.2% vs. 5.4%), cognitive impairment (1.9% vs. 0.5%), and co-occurrence of the two (1.2% vs. 0.1%). Frailty was related to global cognitive function and all cognitive domains, and these associations did not differ by CKD (all p for interactions > 0.5). Frailty did not improve mortality prediction among those without CKD, but did among those with CKD (HR = 2.74, 95%CI: 1.74, 4.32; p for LR < 0.001; C-stat = 0.751). The predictive ability of cognitive impairment was negligible for both those with and without CKD.The distribution of frailty, cognitive impairment, and co-occurrence burden is well described. Among the National Health and Aging Trends Study (NHATS), another nationally-representative study of older adults covered by Medicare, older adults overall had a high prevalence of cognitive impairment only (25.5%), frailty only (5.6%), and co-occurrence (8.7%) [67]. Notably, this NHATS study used different cognitive tests to define cognitive impairment, and included an older population of retirement age (≥ 65 years) on Medicare. Our study expands upon those prior findings by using a more diverse, nationally representative population of older adults aged 60 years and older not limited by Medicare coverage, and by quantifying the burden among those with CKD, a vulnerable population. Importantly, those with CKD had a much greater burden of frailty (13.2% vs. 5.4%), cognitive impairment (1.9% vs. 0.5%), and co-occurrence of the two (1.2% vs. 0.1%) compared to those without CKD. This finding is likely due to differences in the availability of measures for both frailty and cognitive impairment compared to prior studies, and the fundamental differences in source populations. Nonetheless, there is face validity in this study’s findings; it was expected that those with CKD would have a greater burden of frailty, cognitive impairment, and co-occurrence of the two conditions; given their higher prevalence of comorbidities, particularly in vascular disease. Further studies should aim to replicate these findings among those with and without CKD in other, diverse population studies with validated measures of frailty and cognitive function to better compare the magnitude of burden.This diverse, nationally-representative study further corroborates the potential vascular underpinnings of the frailty-cognition link demonstrated in prior studies [27–30, 35]. Our findings support those prior studies in that the strongest associations between frailty and cognitive function were found for executive function and processing speed (Cohen’s d = -0.44, 95%CI: -0.60, -0.28) compared to other memory-related domains. Our study found that CKD did not synergistically affect the relationship between frailty and cognitive performance (all p for interactions > 0.05), despite prior findings demonstrating a link between reduced kidney function and lower cognitive performance; especially in executive function [68–71]. Our analysis dichotomized CKD by defining its presence as eGFR < 60 mL/min/1.73m^2^ or ACR ≥ 30 mg/g; it may be that a future analysis using eGFR measures more granularly, rather than presence of CKD, could uncover a potential interaction at a different threshold of eGFR.While frailty did not improve the prediction of mortality among those without CKD, it did improve predictive performance among those with CKD compared to a base model (HR = 2.74, 95%CI: 1.74, 4.32; p for LR < 0.001; C-stat = 0.751). These results are in line with findings from a systematic review of frailty and CKD which found that frailty was a significant predictor of adverse health outcomes, particularly in those with severe CKD stages [72]. Though the relationship between frailty and CKD is not completely understood, studies have found that inflammation is associated with frailty in many chronic diseases, which suggests a potential “shared pathophysiology” of frailty [72, 73]. However, the causal relationship between inflammation and frailty, specifically in patients with CKD, is yet to be characterized [72]; but may provide critical understanding for approaches to improve patient survival. Clinicians may consider screening people with CKD for frailty, to identify these who are at risk for higher mortality.Additionally, compared to baseline models, the predictive ability of cognitive impairment was negligible for those with CKD (p for LR = 0.19) and those without CKD (p for LR = 0.90). This result may be because people with cognitive impairment might be living longer with such impairments [74] with advancements in therapeutics of symptom management. Further, cognitive function is a continuum; Activities of Daily Living (ADLs) functions are only impacted at later stages of the disease [75]. It may be that people with CKD are dying from other cardiovascular-related causes before they progress to moderate to severe cognitive impairment. Future studies should investigate this further in populations with greater sample sizes and comprehensive collections of cognitive impairment measures; it may be beneficial to explore cognitive scores categorically in order to uncover mortality risk among individuals with the highest levels of cognitive impairment.Several notable limitations deserve comment. Most importantly, the cross-sectional nature of the data does not allow for any understanding of the temporality of the onset of these two conditions; the relationship between CKD, frailty, and cognitive function should be studied prospectively. Additionally, as an observational study, NHANES is subject to unmeasured confounding factors. Though we adjusted for many known correlates of kidney impairment, frailty, and cognitive function, the same individuals who tend to have frailty and CKD may be more vulnerable to having reduced cognitive performance due to unmeasured confounders. Despite the comprehensive nature of NHANES data collection, many participants were missing a cognitive assessment, which may have impacted the associations between cognitive impairment and mortality or not allowed us to pick up important relationships. In addition, as muscle mass decreases, calculated eGFR may falsely appear in a healthy range in older adults; thus, measures of eGFR may not appropriately capture early stage CKD. Finally, operationalization of frailty, in particular, was limited by the availability of measures unique to NHANES; for example, we did not have an objective measure of gait speed. However, any misclassification resulting from specific measures used to define frailty in NHANES is likely to have been non-differential (regardless of true frailty status), so results presented herein are likely to be conservative.Despite these limitations, this study has many strengths. It extends prior findings to a large, novel, nationally-representative sample of individuals aged ≥ 60 years. It additionally presents objective measures of cognitive function spanning multiple key cognitive domains, as well as key measures of physical function which allowed us to adapt and operationalize the physical frailty phenotype; the most widely used measure of frailty both in etiological and clinical research [76].

## Conclusions

In conclusion, frailty is associated with worse cognitive function regardless of CKD status. While frailty improved mortality prediction among those with CKD, cognitive impairment did not. With growing, increasingly diverse aging populations, geriatricians face challenges in accurately prognosticating risks for their older patients. In efforts to support a precision medicine paradigm, risk prediction tools should be made appropriate for older adults generally, and for older adults with highly prevalent conditions like CKD. As such, risk prediction tools should consider incorporating frailty to improve mortality prediction among those with CKD specifically.

### Supplementary Information

Supplementary Material 1.

## Data Availability

The NHANES dataset(s) supporting the conclusions of this article are publicly available at [https://wwwn.cdc.gov/nchs/nhanes/]. Analytic code will be made available upon request.

## Abbreviations

ACR: Serum creatinine and albuminuria
ADL: Activities of daily living
AF: Animal fluency
AIC: Akaike’s information criterion
BIC: Bayesian information criterion
BMI: Body mass index
CERAD: Consortium to Establish a Registry for Alzheimer’s Disease
CHD: Coronary heart disease
CKD: Chronic kidney disease
DBP: Diastolic blood pressure
DSST: Digit Symbol Substitution Test
eGFR: Estimated glomerular filtration rate
ESKD: End-stage kidney disease
IQR: Inter-quartile range
LR: Likelihood ratio
MI: Myocardial infarction
NHANES: National Health and Nutrition Examination Survey
NHATS: National Health and Aging Trends Study
PHQ: Patient Health Questionnaire
SD: Standard deviation
SPH: Systolic blood pressure
