# Supplementary material for: Frailty, but not cognitive impairment, improves mortality risk prediction among those with chronic kidney disease—a nationally representative study
Source: BMC Nephrol. 2024 May 22;25:177. doi: 10.1186/s12882-024-03613-y (PMC11112880; doi:10.1186/s12882-024-03613-y)

**SUPPLEMENTARY MATERIAL**

**Supplementary Table 1. Adapted physical frailty phenotype criteria in the National Health and Nutrition Examination Survey (NHANES) (2011-2014).** Participants were scored as 0 (absence) or 1 (presence) for each of the five physical frailty phenotype criteria. Scores were summed to create a frailty score ranging from 0-5. Participants with a score of 3-5 were defined as frail and 0-2 were defined as not frail.

| Frailty component | Criteria |
| --- | --- |
| Weight loss | (1) BMI < 18.5 kg/m^2^, or  (2) self-report of ≥ 10 pounds of weight loss in the previous year, or  (3) self-reported ≥ 5% loss of body weight in the previous year. |
| Weakness | Grip strength (kg) in the lowest 20%, adjusted for sex and BMI quartiles. |
| Exhaustion | Self-reported of at least one of the following criteria on more than half of the days or nearly every day in the last 2 weeks:  (1) little interest or pleasure in doing things, or  (2) feeling tired or having little energy. |
| Slowness | Self-reported difficulty in walking from one room to another on the same level. |
| Low physical activity | Self-reported physical activity (NHANES metabolic equivalent task (MET) score) in the lowest 20%, adjusted for sex. |

**Supplementary Table 2. Unadjusted association between frailty, chronic kidney disease (CKD) and global and domain-specific cognitive function among participants aged 60 years and older from the National Health and Nutrition Examination Survey (NHANES) (2011-2014) (n=3,211).** Cognitive test scores were standardized to a mean of 0 and standard deviation of 1. Global cognitive function was defined as average score of all 4 objective cognitive tests. NHANES sampling weights were accounted for in linear regression analyses to obtain nationally-representative estimates. eGFR was calculated using serum creatinine and the CKD-EPI equation; CKD was defined as eGFR < 60 mL/min/1.73m^2^ or ACR ≥ 30 mg/g. Frailty was categorized using an adapted5-item frailty phenotype. Abbreviations: CI, confidence interval; CERAD-WL, the Consortium to Establish a Registry for Alzheimer’s Disease word learning subtest immediate recall module; CERAD-DR, the Consortium to Establish a Registry for Alzheimer’s Disease word learning subtest delayed recall module; AF, the Animal Fluency test; DSST, the Digit Symbol Substitution test.

|  | Overall | No CKD | CKD |  |
| --- | --- | --- | --- | --- |
|  | Mean (95% CI) | Mean (95% CI) | Mean (95% CI) | p for interaction |
| Global cognitive function |  |  |  |  |
| Not frail | 0 (reference) | 0 (reference) | 0 (reference) |  |
| Frail | -0.55 (-0.68, -0.42) | -0.43 (-0.64, -0.23) | -0.50 (-0.64, -0.36) | 0.58 |
| Immediate recall (CERAD-WL) |  |  |  |  |
| Not frail | 0 (reference) | 0 (reference) | 0 (reference) |  |
| Frail | -0.50 (-0.63, -0.37) | -0.44 (-0.68, -0.20) | -0.41 (-0.57, -0.25) | 0.84 |
| Delayed recall (CERAD-DR) |  |  |  |  |
| Not frail | 0 (reference) | 0 (reference) | 0 (reference) |  |
| Frail | -0.45 (-0.56, -0.33) | -0.36 (-0.59, -0.13) | -0.39 (-0.59, -0.20) | 0.86 |
| Verbal fluency (AF) |  |  |  |  |
| Not frail | 0 (reference) | 0 (reference) | 0 (reference) |  |
| Frail | -0.65 (-0.82, -0.48) | -0.51 (-0.76, -0.27) | -0.62 (-0.77, -0.48) | 0.38 |
| Executive function & processing speed (DSST) |  |  |  |  |
| Not frail | 0 (reference) | 0 (reference) | 0 (reference) |  |
| Frail | -0.84 (-1.03, -0.64) | -0.71 (-0.93, -0.49) | -0.75 (-0.97, -0.53) | 0.78 |

**Supplementary Table 3. Association between frailty, chronic kidney disease (CKD) and self-perceived cognitive decline** **among participants aged 60 years and older from the National Health and Nutrition Examination Survey (NHANES) (2011-2014) (n=3,211).** Models were adjusted for age, sex, race, education, hypertension, diabetes, coronary heart disease, myocardial infarction, stroke, anemia, and smoking. NHANES sampling weights are accounted for in logistic regression analysis to obtain nationally-representative estimates. EGFR was calculated using serum creatinine and the CKD-EPI equation; CKD was defined as eGFR < 60 mL/min/1.73m^2^ or ACR ≥ 30 mg/g. Frailty was categorized using an adapted, 5-item frailty phenotype. Abbreviations: CI, confidence interval; CKD, chronic kidney disease; OR, odds ratio.

| Subjective cognitive function | Overall | No CKD | CKD |  |
| --- | --- | --- | --- | --- |
|  | OR (95% CI) | OR (95% CI) | OR (95% CI) | p for interaction |
| Not frail | 1 (reference) | 1 (reference) | 1 (reference) |  |
| Frail | 2.60 (1.81, 3.75) | 3.24 (1.99, 5.30) | 2.07 (1.39, 3.09) | 0.09 |

**Supplementary Table 4. Association between 4-item frailty, chronic kidney disease (CKD) and global and domain-specific cognitive function among participants aged 60 years and older from the National Health and Nutrition Examination Survey (NHANES) (2011-2014) (n=3,211).** Cognitive test scores were standardized to a mean of 0 and standard deviation of 1. Global cognitive function was defined as average score of all 4 objective cognitive tests. Models were adjusted for age, sex, race, education, hypertension, diabetes, coronary heart disease, myocardial infarction, stroke, anemia, and smoking. NHANES sampling weights were accounted for in linear regression analyses to obtain nationally-representative estimates. EGFR was calculated using serum creatinine and the CKD-EPI equation; CKD was defined as eGFR < 60 mL/min/1.73m^2^ or ACR ≥ 30 mg/g. Frailty was categorized using an adapted 4-item frailty phenotype (weight loss, weakness, slowness, low physical activity). Abbreviations: CI, confidence interval; CERAD-WL, the Consortium to Establish a Registry for Alzheimer’s Disease word learning subtest immediate recall module; CERAD-DR, the Consortium to Establish a Registry for Alzheimer’s Disease word learning subtest delayed recall module; AF, the Animal Fluency test; DSST, the Digit Symbol Substitution test.

|  | Overall | No CKD | CKD |  |
| --- | --- | --- | --- | --- |
|  | Mean (95% CI) | Mean (95% CI) | Mean (95% CI) | p for interaction |
| Global cognitive function |  |  |  |  |
| Not frail | 0 (reference) | 0 (reference) | 0 (reference) |  |
| Frail | -0.18 (-0.29, -0.07) | -0.12 (-0.30, 0.07) | -0.24 (-0.41, -0.07) | 0.40 |
| Immediate recall (CERAD-WL) |  |  |  |  |
| Not frail | 0 (reference) | 0 (reference) | 0 (reference) |  |
| Frail | -0.20 (-0.34, -0.07) | -0.23 (-0.53, 0.07) | -0.16 (-0.34, 0.02) | 0.74 |
| Delayed recall (CERAD-DR) |  |  |  |  |
| Not frail | 0 (reference) | 0 (reference) | 0 (reference) |  |
| Frail | -0.08 (-0.19, 0.03) | -0.08 (-0.34, 0.17) | -0.07 (-0.30, 0.16) | 0.95 |
| Verbal fluency (AF) |  |  |  |  |
| Not frail | 0 (reference) | 0 (reference) | 0 (reference) |  |
| Frail | -0.26 (-0.44, -0.08) | -0.19 (-0.45, 0.06) | -0.32 (-0.52, -0.12) | 0.41 |
| Executive function & processing speed (DSST) |  |  |  |  |
| Not frail | 0 (reference) | 0 (reference) | 0 (reference) |  |
| Frail | -0.37 (-0.56, -0.18) | -0.29 (-0.53, -0.05) | -0.44 (-0.67, -0.22) | 0.31 |

**Supplementary Table 5. Details of follow-up among participants aged 60 years and older from the National Health and Nutrition Examination Survey (NHANES) (2011-2014) (n=3,211).** Number of persons at risk and number of events are presented. Participants were followed for a maximum of 61 months, with the median follow-up time of 30 months. The outcome was all-cause death. eGFR was calculated using serum creatinine and the CKD-EPI equation; CKD was defined as eGFR < 60 mL/min/1.73m^2^ or ACR ≥ 30 mg/g. Frailty was categorized using an adapted 5-item frailty phenotype.

| CKD | Frailty | Persons at risk | Events |
| --- | --- | --- | --- |
| No CKD | Not frail | 1891 | 64 |
| No CKD | Frail | 156 | 14 |
| CKD | Not frail | 949 | 113 |
| CKD | Frail | 211 | 64 |

**Supplementary Figure 1. Cumulative hazard of mortality by CKD and frailty among participants aged 60 years and older in the National Health and Nutrition Examination Survey (NHANES) (2011-2014) CKD (n=3,211).** NHANES sampling weights were accounted for to obtain nationally-representative estimates. EGFR was calculated using serum creatinine and the CKD-EPI equation; CKD was defined as eGFR < 60 mL/min/1.73m^2^ or ACR ≥ 30 mg/g. Frailty was categorized using an adapted 5-item physical frailty phenotype.

**Supplementary Figure 2. Distributions of objective cognitive test scores over chronic kidney disease (CKD) among participants aged 60 years and older from the National Health and Nutrition Examination Survey (NHANES) (2011-2014) (n=3,211).** Medians and inter-quartile ranges (IQRs) are presented for objective cognitive test scores, which were standardized to mean of 0 and standard deviation of 1. Global cognitive function was defined as average score of all 4 objective cognitive tests. NHANES sampling weights were accounted for to obtain nationally-representative estimates. eGFR was calculated using serum creatinine and the CKD-EPI equation; CKD was defined as eGFR < 60 mL/min/1.73m^2^ or ACR ≥ 30 mg/g. Abbreviations: CERAD-WL, the Consortium to Establish a Registry for Alzheimer’s Disease word learning subtest immediate recall module; CERAD-DR, the Consortium to Establish a Registry for Alzheimer’s Disease word learning subtest delayed recall module; AF, the Animal Fluency test; DSST, the Digit Symbol Substitution test.

**Supplementary Figure 3. Distributions of objective cognitive test scores over chronic kidney disease (CKD) and frailty among participants aged 60 years and older from the National Health and Nutrition Examination Survey (NHANES) (2011-2014) (n=3,211).** Medians and inter-quartile ranges (IQRs) are presented for objective cognitive test scores, which were standardized to mean of 0 and standard deviation of 1. Global cognitive function was defined as average score of all 4 objective cognitive tests. NHANES sampling weights were accounted for to obtain nationally-representative estimates. EGFR was calculated using serum creatinine and the CKD-EPI equation; CKD was defined as eGFR < 60 mL/min/1.73m^2^ or ACR ≥ 30 mg/g. Frailty was categorized using an adapted 5-item frailty phenotype. Abbreviations: CERAD-WL, the Consortium to Establish a Registry for Alzheimer’s Disease word learning subtest immediate recall module; CERAD-DR, the Consortium to Establish a Registry for Alzheimer’s Disease word learning subtest delayed recall module; AF, the Animal Fluency test; DSST, the Digit Symbol Substitution test.

**Supplementary Figure 4. Cumulative hazard of mortality by frailty and cognitive impairment among participants aged 60 years and older with and without CKD from the National Health and Nutrition Examination Survey (NHANES) (2011-2014) (n=2,663).** Cumulative hazard graphs are presented in accordance with predictive models of CKD, frailty, and/or cognitive impairment on risk of mortality in Table 3, adjusting for age, sex, race, education, hypertension, diabetes, coronary heart disease, myocardial infarction, stroke, anemia, and smoking. EGFR was calculated using serum creatinine and the CKD-EPI equation; CKD was defined as eGFR < 60 mL/min/1.73m^2^ or ACR ≥ 30 mg/g. Frailty was categorized using an adapted 5-item physical frailty phenotype. Cognitive impairment was defined as global cognitive function (average score of all 4 objective cognitive tests) less than 1.5 standard deviation below the mean.

**Supplementary Figure 1.**


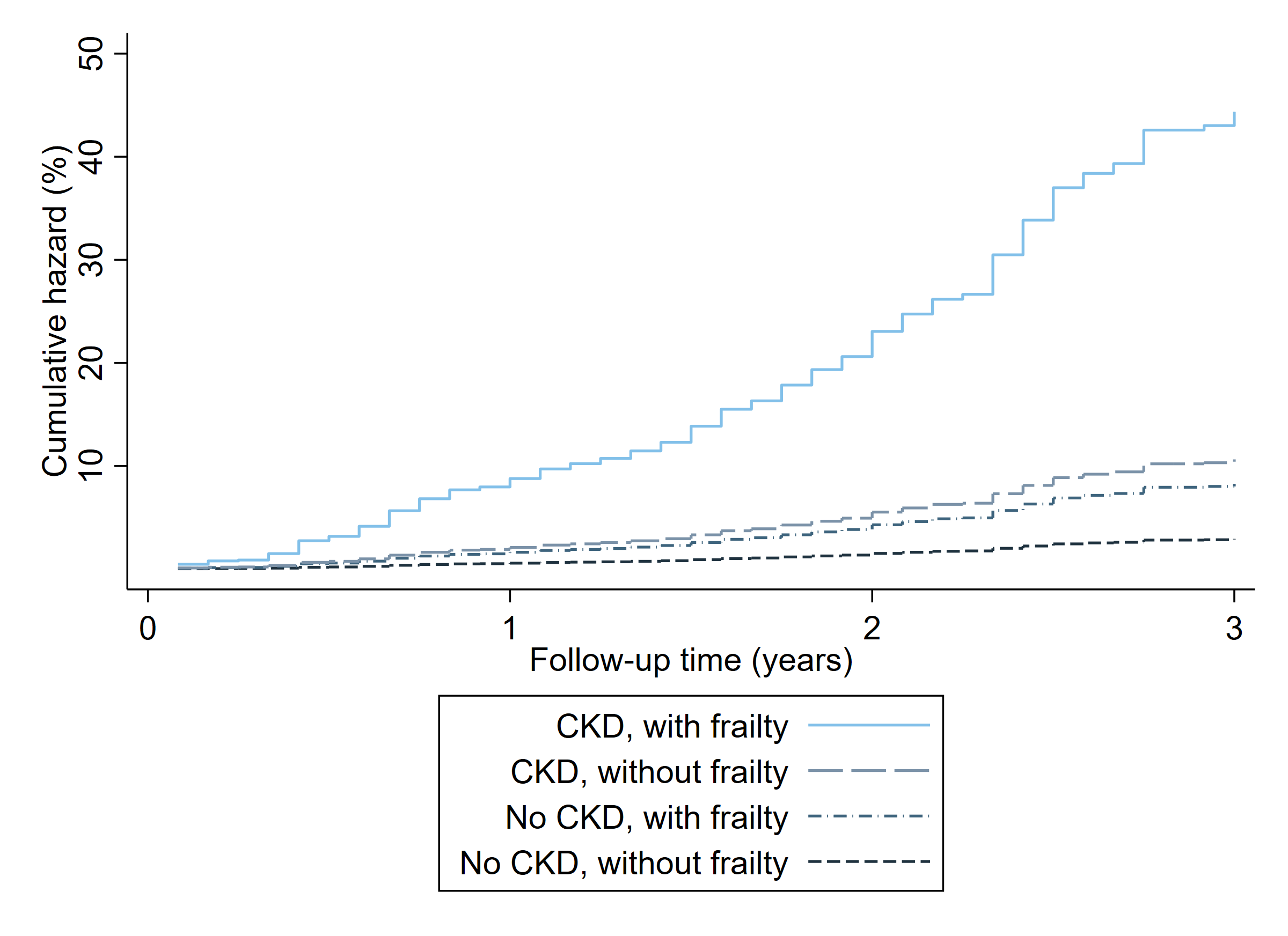


**Supplementary Figure 2.**


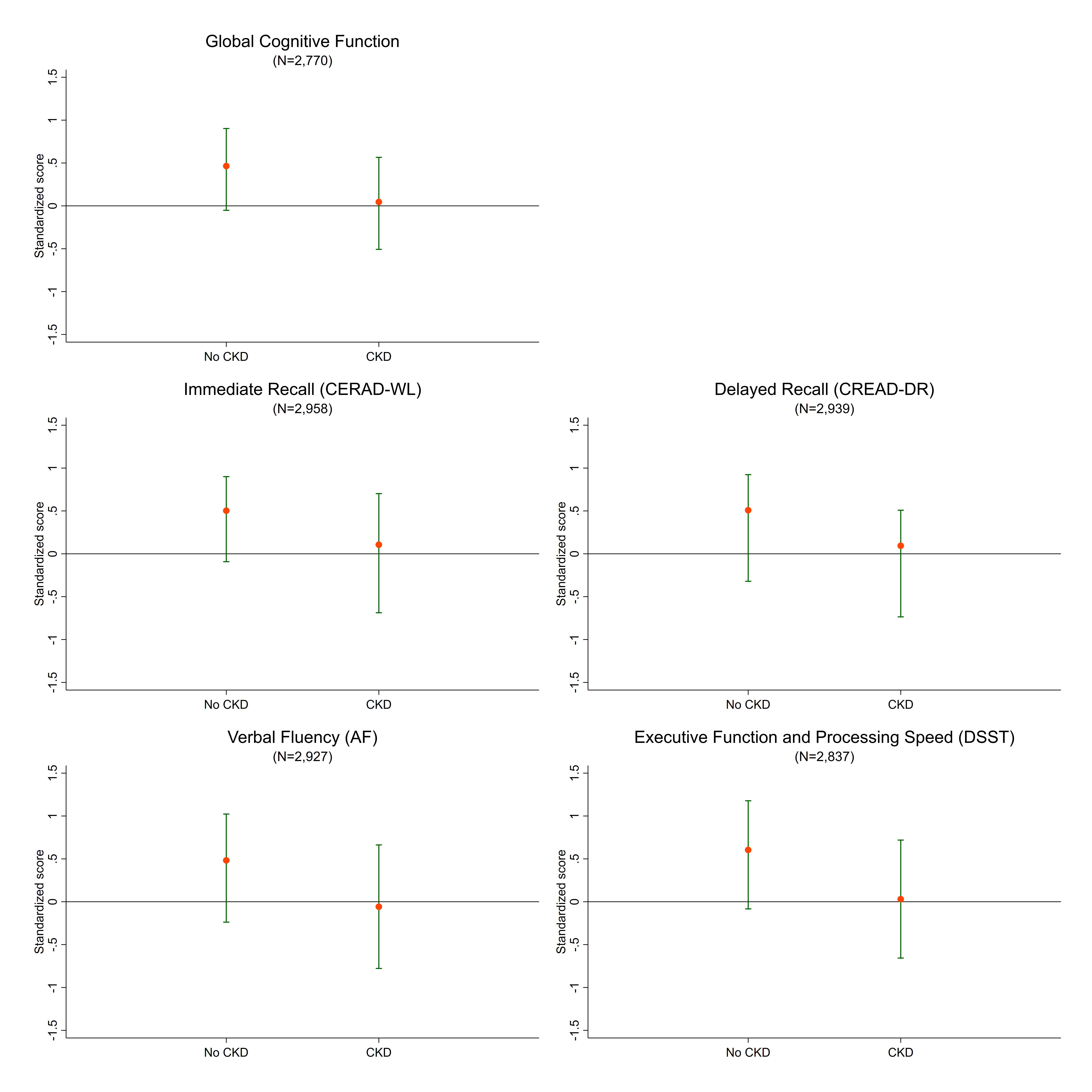


**Supplementary Figure 3.**


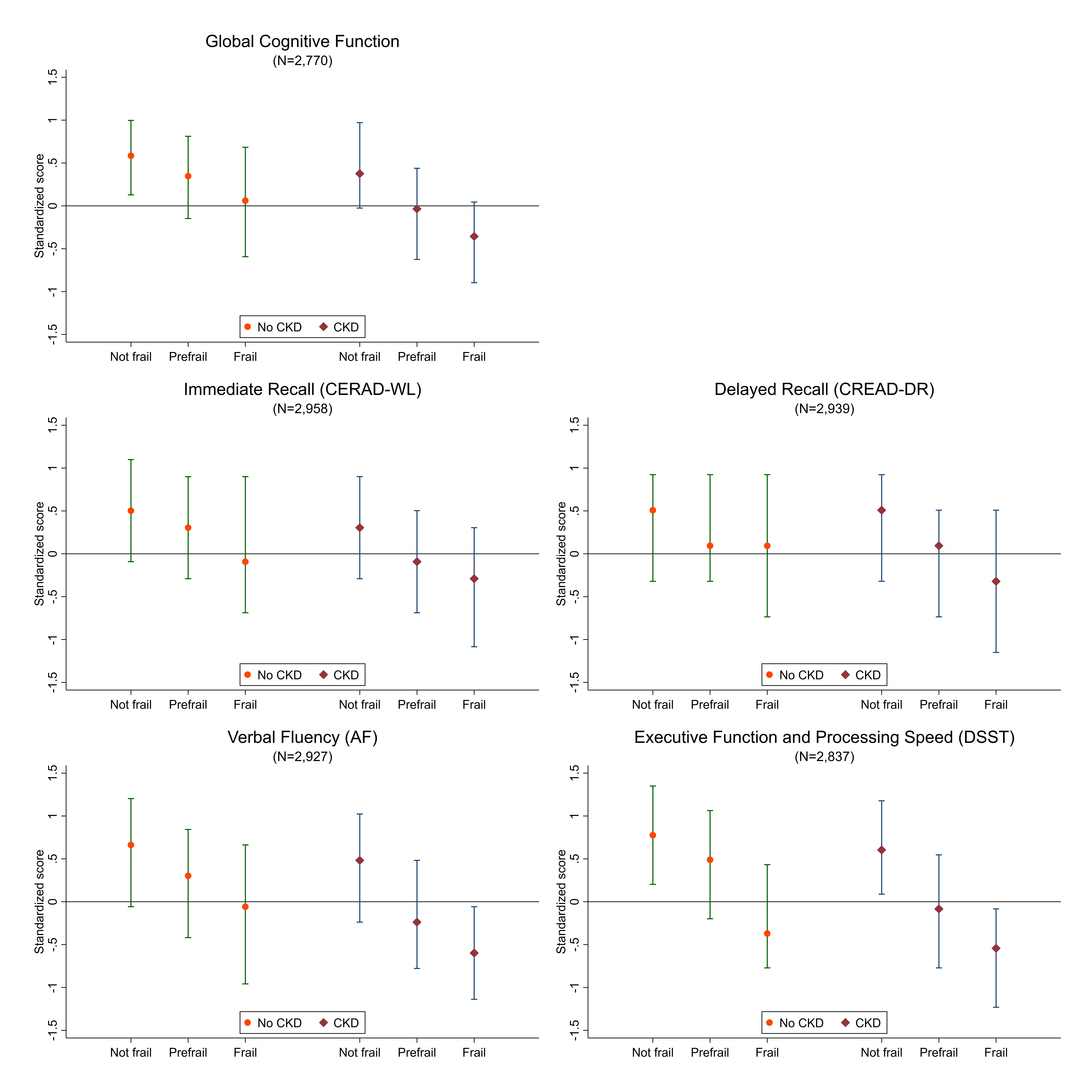


**Supplementary Figure 4.**


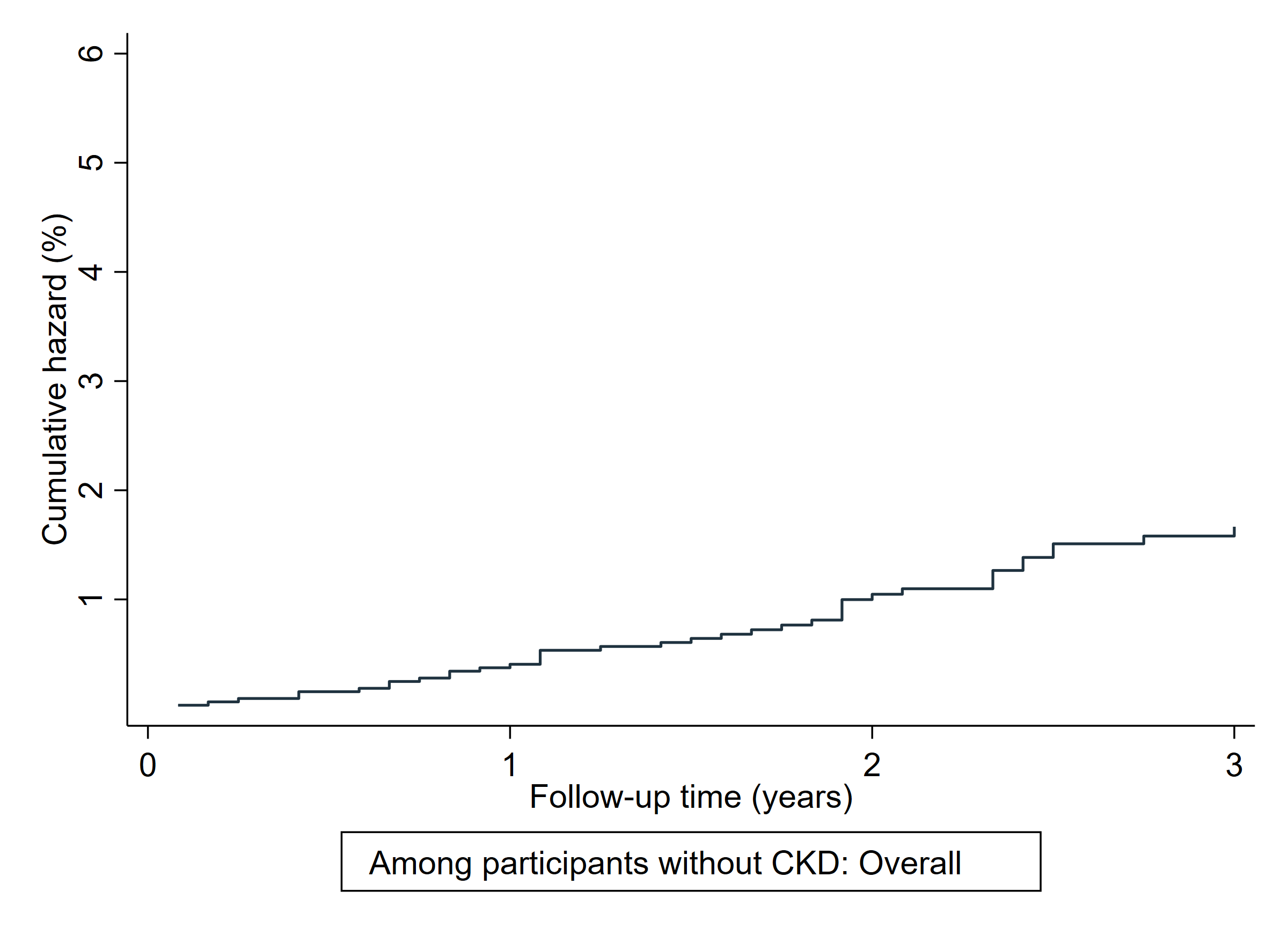

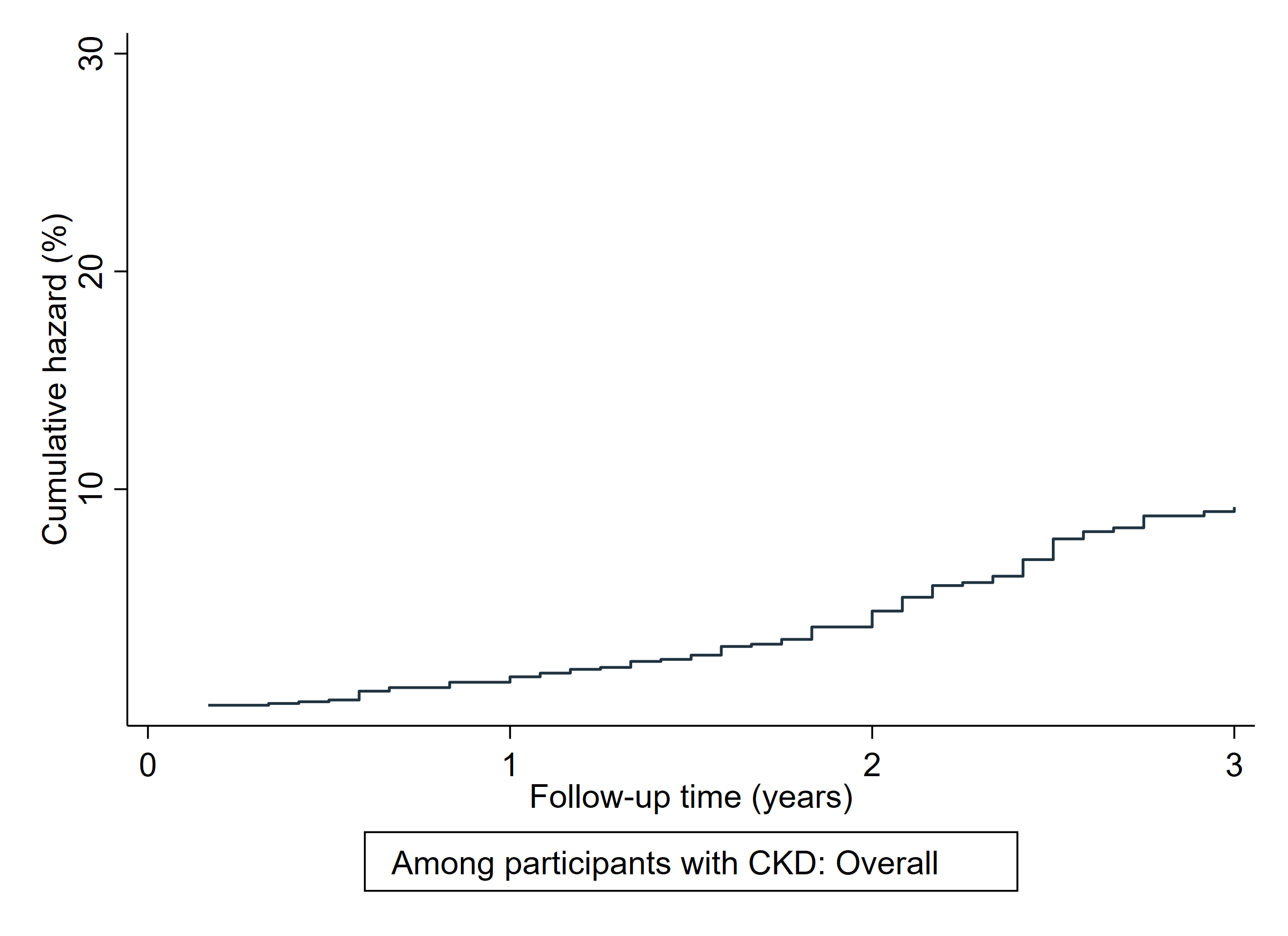


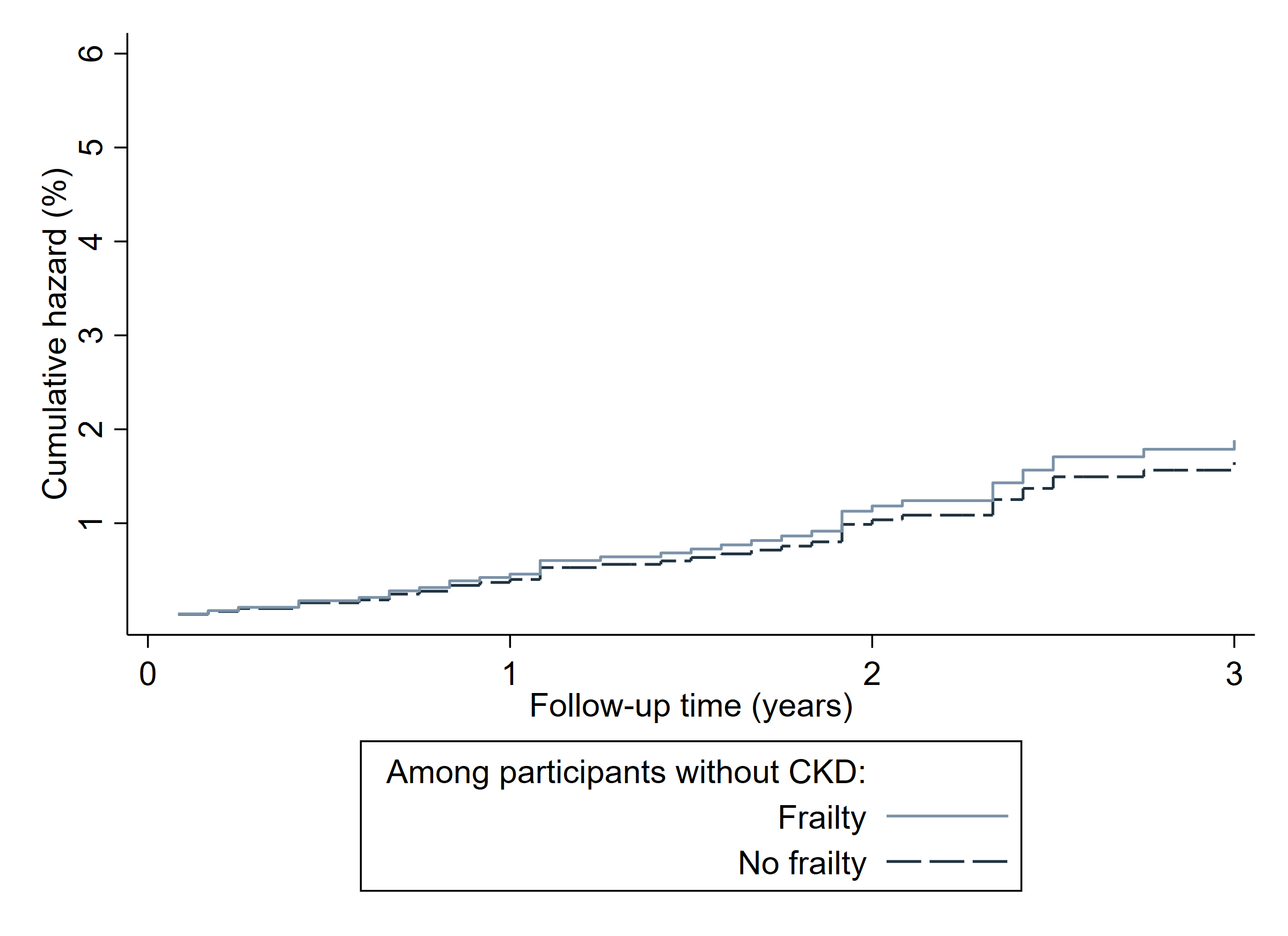

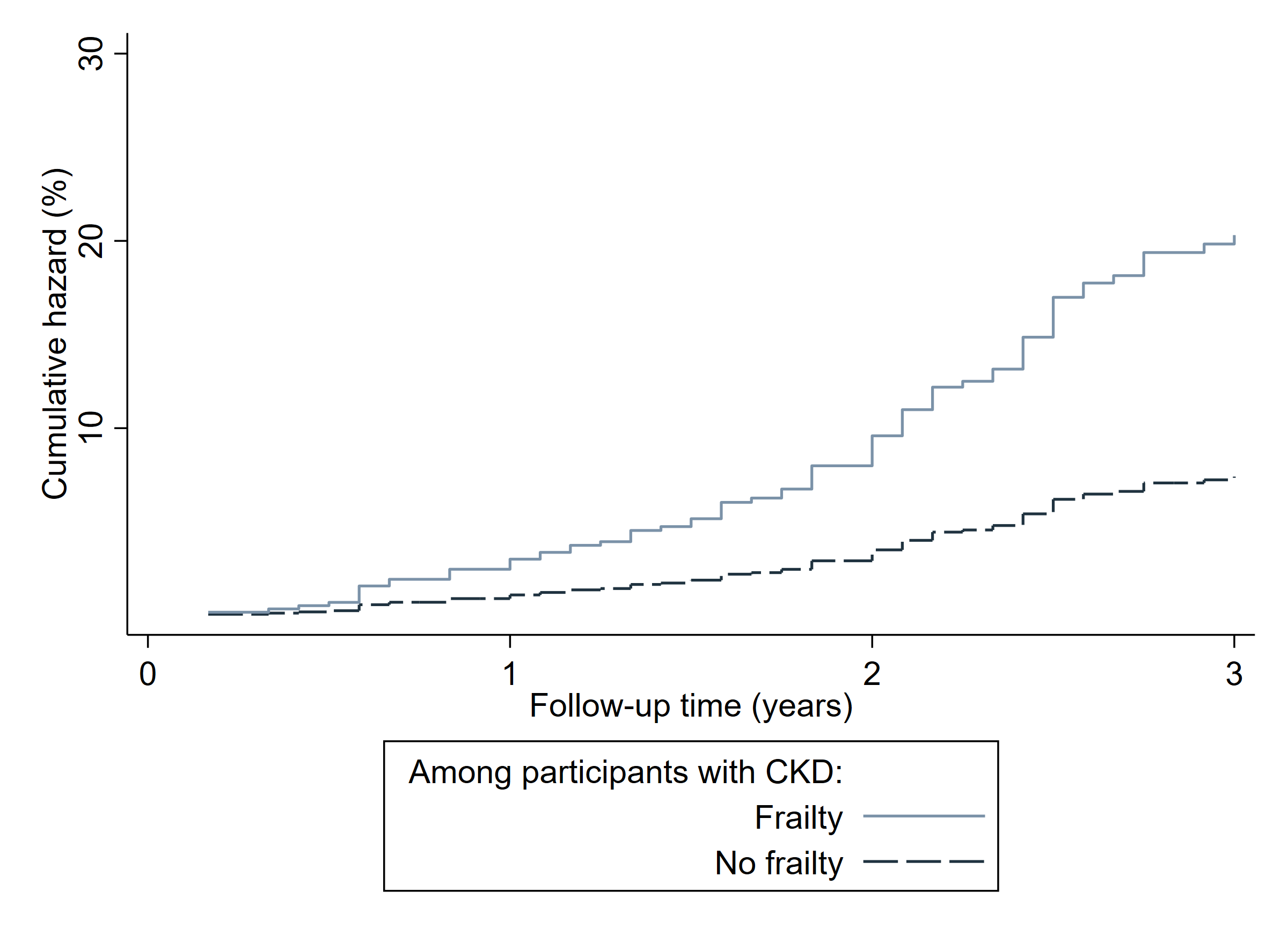


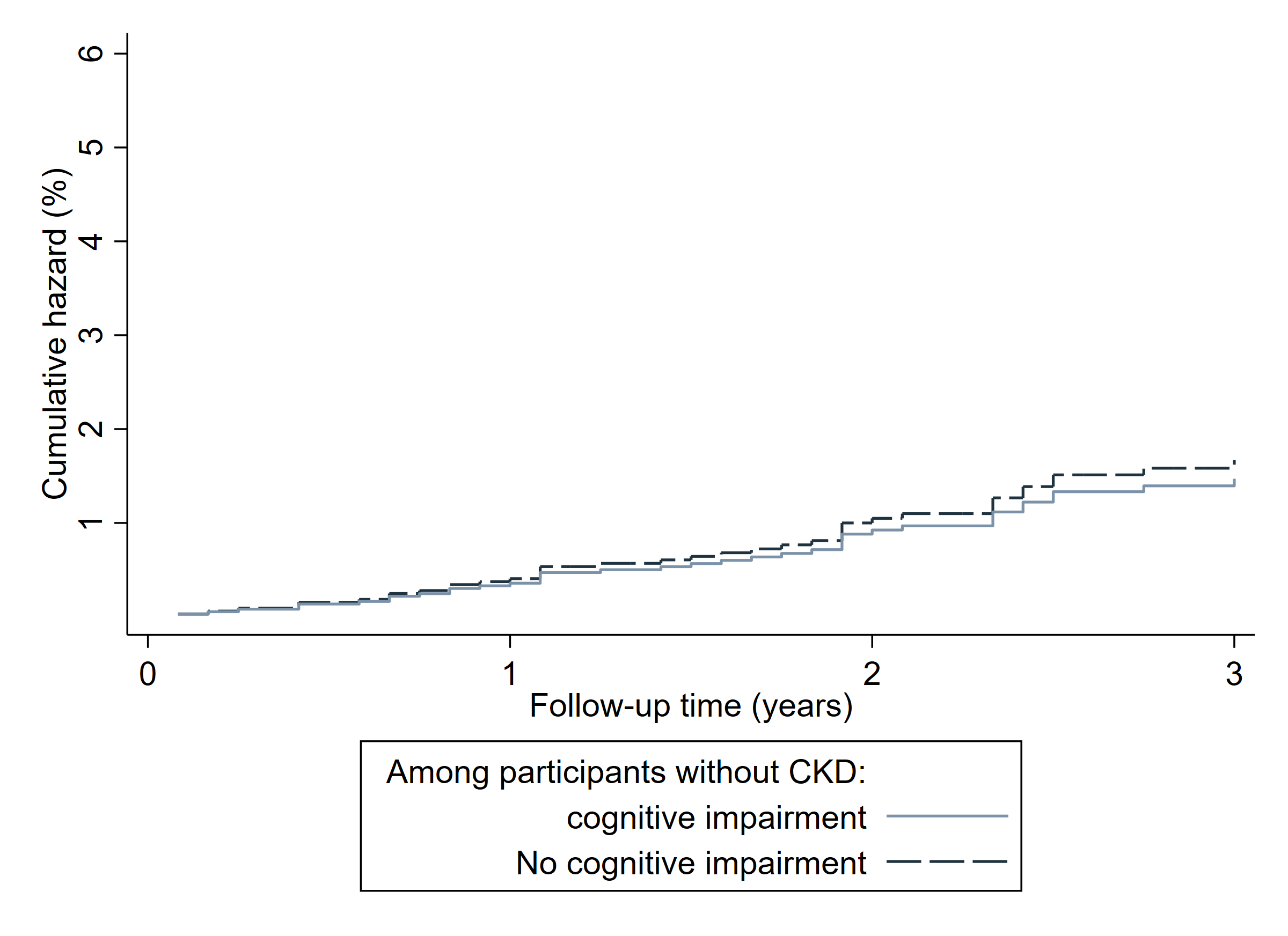

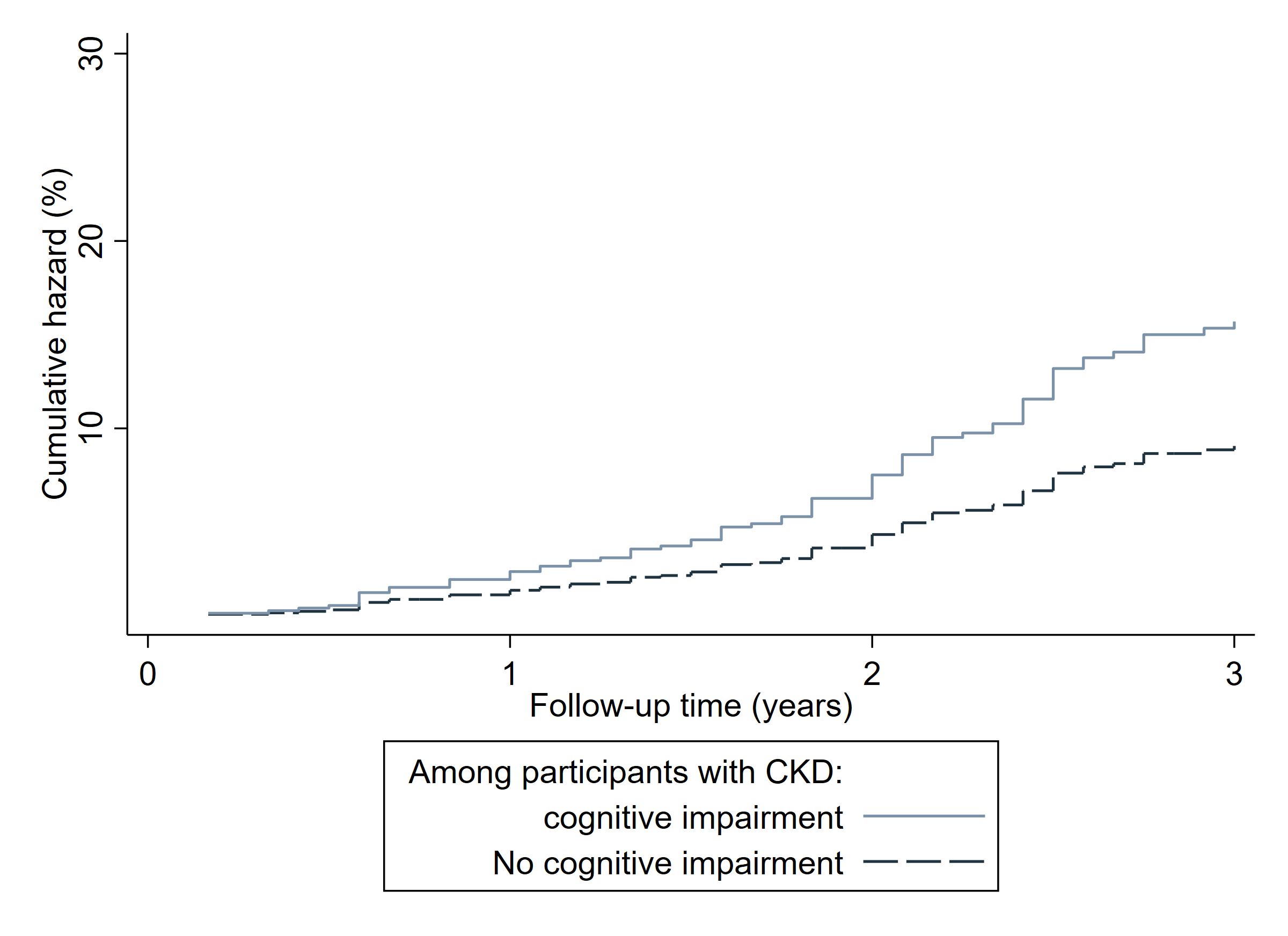


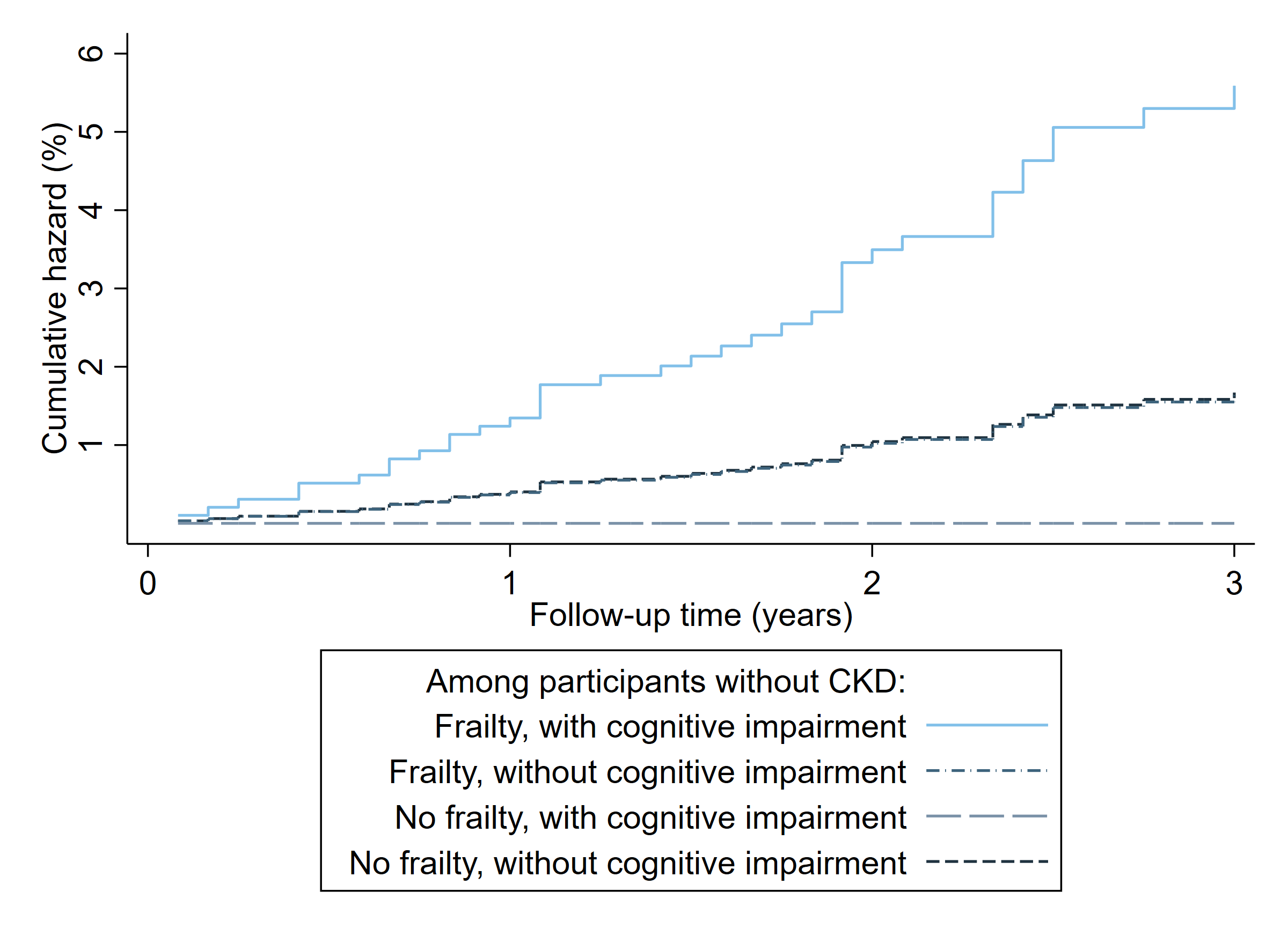

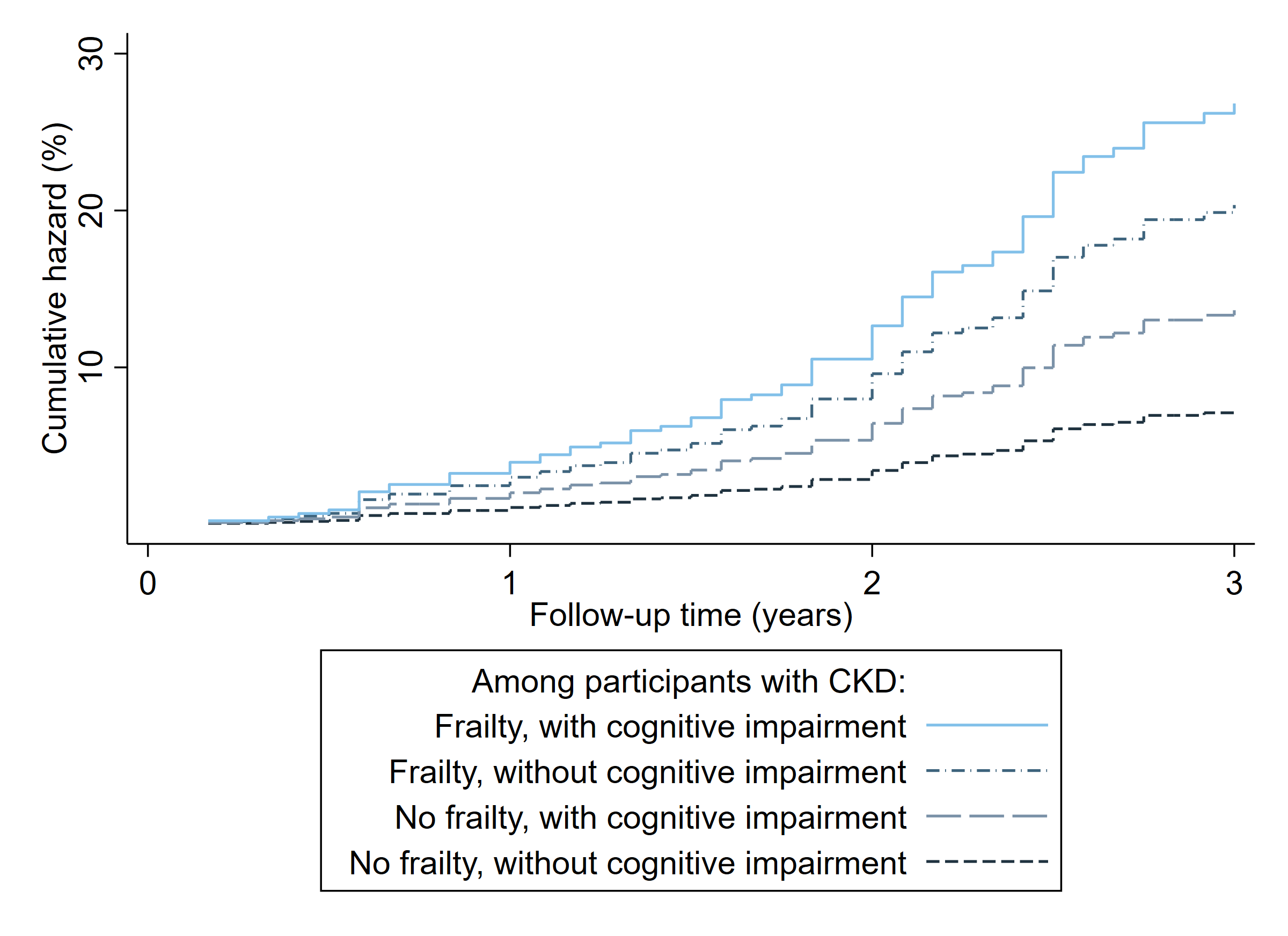

Supplement: Supplementary file 1 — Supplementary Material 1. [file 12882_2024_3613_MOESM1_ESM.docx]
